# Successful treatment of hidradenitis suppurativa with vunakizumab: a case report and literature review

**DOI:** 10.3389/fimmu.2026.1852135

**Published:** 2026-07-29

**Authors:** Mei Zhang, Ming He, Ting Tang

**Affiliations:** 1 The First Clinical Medical College, Guizhou University of Traditional Chinese Medicine, Guiyang, China; 2 Department of Dermatology, The First Affiliated Hospital of Guizhou University of Traditional Chinese Medicine, Guiyang, China

**Keywords:** case report, hidradenitis suppurativa, IL-17 inhibitor, pharmacoeconomics, vunakizumab

## Abstract

Hidradenitis suppurativa (HS) is a chronic, recurrent, and disabling inflammatory skin disorder. To our knowledge, this study reports the first use of vunakizumab, a domestically developed anti-IL-17A monoclonal antibody, for the treatment of HS. We present the case of a 17-year-old boy with a 5-year history of recurrent painful nodules and abscesses on the buttocks who had been repeatedly misdiagnosed with common acute bacterial infections and had responded poorly to conventional antibiotics. After 8 weeks of treatment with vunakizumab, which offered a pharmacoeconomic advantage during the transition period before its inclusion in China’s national insurance system, the patient showed marked improvement, with complete resolution of nodules, abscesses, and tunnels and no recurrence or significant adverse events during 10 months of follow-up. This case highlights the potential of vunakizumab in the treatment of HS and supports the pathogenic role of IL-17 in the disease.

## 1 Introduction

Hidradenitis suppurativa (acne inversa) is a chronic, recurrent inflammatory skin disease resulting from follicular occlusion that affects the pilosebaceous unit. It predominantly involves apocrine gland-rich areas such as the axillae, groin, perineum, and perianal region and presents with painful, deep-seated inflammatory nodules that progress to abscesses, tunnels, and scarring (1). Patients experience a significantly reduced quality of life because of severe pain, purulent discharge, restricted movement, and systemic comorbidities (e.g., metabolic syndrome, inflammatory arthritis, and inflammatory bowel disease) (2). The pathogenesis of HS is complex and multifactorial, involving genetic susceptibility, immune dysregulation, and microbial and lifestyle factors (2). Therefore, treatment remains challenging. Conventional strategies include lifestyle modifications, topical and systemic antibiotics, intralesional corticosteroid injections, traditional immunosuppressants, and surgery (3, 4). However, these therapies often fail to achieve adequate disease control in patients with moderate-to-severe HS. With increasing insights into immune dysregulation in HS, a growing number of biological agents and small-molecule targeted drugs have been used to treat moderate-to-severe HS (5).

The IL-17 pathway plays a key role in HS pathogenesis and has recently emerged as a therapeutic target (6). Vunakizumab, a domestically developed anti-IL-17A monoclonal antibody, is a potentially effective treatment for HS. However, to date, its use in HS has not been reported. To our knowledge, this paper is the first to describe a case of a 17-year-old patient with moderate-to-severe HS who achieved rapid clinical improvement with vunakizumab and maintained good efficacy and safety over 10 months of follow-up, after years of misdiagnosis and disease recurrence. Additionally, we reviewed key clinical studies on IL-17 inhibitors for HS published in the past five years.

## 2 Case report

A 17-year-old boy presented to our department in March 2025 with a 5-year history of recurrent painful nodules and abscesses on the buttocks that had worsened over the past week. His parents reported that since the age of 12 years, he had experienced three to five episodes per year without obvious triggers, with more than 2 episodes in the preceding six months. He was repeatedly diagnosed with furunculosis, carbuncles, or bacterial infections of the buttocks and treated with oral amoxicillin and topical fusidic acid cream, which provided temporary relief but failed to prevent recurrence and progression, eventually leading to the formation of tunnels and scarring. He was otherwise healthy with no history of smoking or HS and a body mass index of 22.8 kg/m^2^. Dermatological examination revealed extensive brown-to-black hyperpigmentation, scattered depigmentation, buttocks scarring, and multiple tender subcutaneous nodules. On the right buttock, an ulcerated abscess measuring approximately 1.5 cm × 3.5 cm was observed, with purulent discharge, sinus tract formation, surrounding fluctuance, and mildly increased local skin temperature (**Figure 1A**). Wound culture yielded a small amount of *Staphylococcus lugdunensis*. Soft tissue ultrasonography of the right buttock revealed a hypoechoic area consistent with abscess formation. Other investigations, including complete blood count, liver and kidney function tests, chest radiography, and tuberculin testing, yielded normal results. According to the 2024 shortened version of the HS S2k guideline (1), the patient presented with typical skin lesions (nodules, abscesses, and tunnels) in a typical location (buttocks) and a typical disease course (recurrence twice within 6 months); therefore, a diagnosis of hidradenitis suppurativa (Hurley stage II) was confirmed. Baseline scores were as follows: numerical rating scale (NRS) for pain, 7; Dermatology Life Quality Index (DLQI), 18; International Hidradenitis Suppurativa Severity Score System (IHS4), 11; and Hidradenitis Suppurativa Physician Global Assessment (HS-PGA), 3.

**Figure 1:**
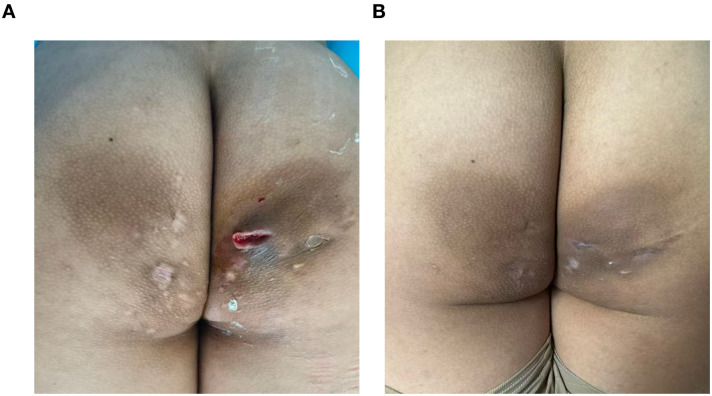
**(A)** Dermatological examination revealed extensive brown-to-black hyperpigmentation, scattered depigmentation, buttocks scarring, and multiple tender subcutaneous nodules. **(B)** After 8 weeks vunakizumab treatment, all painful nodules and the sinus tract had completely resolved, leaving only hyperpigmentation and minor scarring.

Given that the secretion culture suggested secondary infection with *Staphylococcus lugdunensis*, we administered intravenous cefazolin sodium 1.0 g three times daily to improve the wound environment. In addition to abscess incision and drainage and local wound care to promote healing, vunakizumab was initiated after informed consent was obtained from the patient’s guardians and an off-label use agreement was signed. This decision was based on the long disease duration and its significant impact on the patient’s studies and quality of life. The dosing regimen was 240 mg subcutaneously (divided into two 120 mg injections) at weeks 0, 2, and 4, followed by 240 mg every 4 weeks for maintenance. Two weeks after the first injection, pain was markedly reduced and the nodules softened and shrank. At 4 weeks, the painful nodules had further decreased in size, and the sinus tract had almost healed. At 8 weeks, all painful nodules and the sinus tract had completely resolved, leaving only hyperpigmentation and minor scarring (**Figure 1B**). The IHS4 score decreased from 11 to 0, and the patient achieved a HiSCR100. Patient-reported outcomes before and after 8 weeks of vunakizumab use are shown in **Table 1**. Six months after the first injection, the patient remained stable, and the regimen was adjusted to alternate between 120 and 240 mg every 4 weeks. At the last follow-up (10 months after treatment initiation), no new lesions appeared, and the quality of life had significantly improved. No common adverse events associated with IL-17 inhibitors, such as oral candidiasis or upper respiratory tract infections, occurred during treatment or follow-up. Regular monitoring of blood counts and liver and kidney function revealed normal results.

**Table 1:** Patient-reported and physician-reported outcomes (HiSCR-AN, IHS4 and HS-PGA) before and after 8 weeks of vunakizumab treatment.

Time point	Pain (NRS)	DLQI	HiSCR-AN	IHS4	HS-PGA
Before treatment	7	18	6	11	3
After 8 weeks of treatment	0	1	0	0	0

NRS, numerical rating scale; DLQI, dermatology life quality index; HiSCR AN, hidradenitis suppurativa clinical response sum of abscesses and inflammatory nodules(Specifically referring the abscess and inflammatory nodule count here); IHS4, international hidradenitis suppurativa severity score system; HS-PGA, hidradenitis suppurativa physician’s global assessment scale.

## 3 Discussion

Studies have shown that IL-17A gene expression is increased 30- to 149-fold in HS lesions and is significantly elevated in the asymptomatic skin of patients with HS (7, 8), indicating a pathogenic role of IL-17A in HS. IL-17 activates keratinocytes, inducing the expression and release of pro-inflammatory mediators (such as S100A8, S100A9, and antimicrobial peptides like β-defensin-2) and various chemokines (such as CXCL1, IL-8, and CCL20), thereby recruiting neutrophils, macrophages, and lymphocytes to the lesional site to generate inflammation, while activating innate and adaptive immunity to sustain tissue inflammation (6). In HS lesional skin, Th17 cells, which are the primary producers of IL-17, are also significantly enriched; these cells express the CCL20 receptor, enabling them to migrate to inflammatory sites and enhance immune responses (9, 10). Furthermore, IL-17 stimulates macrophages to produce TGF-β, IL-1β, IL-6, and TNF through mechanisms involving the NLRP3 inflammasome and Th17 cell proliferation, thereby promoting further IL-17 production (7, 11) and inducing keratinocytes and fibroblasts to express additional chemokines (12), creating a persistent vicious cycle of “inflammation exacerbation – tissue damage – cytokine upregulation.” Among these, fibroblasts respond to IL-1β by producing additional chemokines and matrix metalloproteinases (MMPs) (MMP1, MMP3, and MMP10), promoting cyst disintegration and tunnel formation (12). The persistence of IL-17 signaling gradually transforms acute lesions into chronic disease, characterized by actively draining sinus tracts with extensive fibrosis. Therefore, persistent activation of the IL-17 signaling pathway is considered a key step in the inflammatory cascade of HS (13), providing a strong rationale for the clinical use of IL-17 inhibitors in moderate-to-severe HS.

Over the past five years, the level of evidence for IL-17 inhibitors in HS has advanced from case reports and open-label studies to large-scale randomized controlled trials (RCTs). Two phase III RCTs of secukinumab showed that the every-2-week regimen achieved significantly higher HiSCR response rates than that achieved via placebo, with efficacy maintained for 52 weeks. Extension studies further supported its long-term safety and efficacy. The dual IL-17A/IL-17F inhibitor bimekizumab demonstrated good clinical response in phase II and III RCTs, with sustained high remission rates for 2 years. Real-world studies of brodalumab have also demonstrated its efficacy. Preliminary clinical investigations of other IL-17 inhibitors, including CJM112, izokibep, and sonelokimab, have been completed (**Table 2**). These results indicate that IL-17 inhibitors are important treatment options for moderate-to-severe HS. Therefore, within the framework of a validated IL-17A inhibition mechanism, vunakizumab has the potential to represent domestic biologic agents for the treatment of HS by leveraging advantages in accessibility, cost, and potentially optimized dosing regimens.

**Table 2:** Overview of reported key clinical studies on IL-17 inhibitors for HS.

Author	Year	Study type	IL-17 inhibitor	Sample size	Primary efficacy endpoint	Outcome	Adverse events
Glatt, et al. (14)	2021	Multicenter Phase 2 RCT	Bimekizumab	88	HiSCR50/7590 (Week 12)	HiSCR50: 57.3% vs. 26.1% (placebo) HiSCR75: 46% vs. 10% (placebo) HiSCR75: 32% vs. 0% (placebo)	Oral candidiasis
Hunger, et al. (15)	2022	Multicenter Phase 2 RCT	CJM112	66	HS-PGA (Week 16)	32.3% vs. 12.5%	Nasopharyngitis, nausea, diarrhea, and headaches
Osorio-Gomez GF, et al. (16)	2023	Multicenter open-label cohort study	Brodalumab	16	HiSCR50, IHS4-50 (Week 16)	HiSCR50: 50%; IHS4-50: 31.25%	Grade 2 AEs in 3 patients (oral aphthosis, generalized non disabling joint pain, Upper respiratory infections)
Kearney, et al. (17)	2023	Retrospective study	Brodalumab	8	DLQI	DLQI decreased from 20.6 to 16.8; 50% achieved DLQI improvement >4 points	None
Kimball, et al. (18)	2023	Multicenter Phase 3 RCT	Secukinumab	1084	HiSCR50 (Week 16)	Q2W: 42%–45% vs. 31%–34% (placebo); Q4W only reached statistical significance in SUNRISE	Headache (most common), nasopharyngitis, recurrence of HS, upper respiratory tract infection, fungal infectious
Kimball, et al. (19)	2024	Multicenter Phase 3 RCT	Bimekizumab	1014	HiSCR50 (Week 16)	Q2W: 48%–52% vs. 29%–32% (placebo); Q4W only reached statistical significance in BE HEARD II	Oral candidiasis
Mansilla-Polo, et al. (20)	2024	Ambispective observational study	Bimekizumab	40	IHS4, HS-PGA (Week 16)	IHS4 decreased from 27.1 to 15.6 (p<0.001), HS-PGA decreased from 5.1 to 3.2 (p<0.001)	Oral candidiasis
Haselgruber, et al. (21)	2024	Multicenter retrospective study	Secukinumab	67	HiSCR50 IHS4-55 (Week 24)	HiSCR50: 41.79%, IHS4-55: 44.78%	Articular pain and inflammation; oral candidiasis; Crohn’s disease
Donald B, et al. (22)	2024	Phase 2b RCT	Izokibep	180	HiSCR75 (Week 12)	HiSCR75: 57%, HiSCR90: 38%, HiSCR100: 33%	Most common: mild to moderate injection site reactions, 3 serious AE: inflammatory bowel disease, peri-colic abscess/sepsis
Kimball, et al. (23)	2024	Phase 2b RCT	Sonelokimab	234	HiSCR75 (Week 12)	34.8%–43.3% vs. 14.7% (placebo)	Favorable safety profile consistent with other IL-17 inhibitors
Kimball, et al. (24)	2025	Multicenter phase 3 RCT extension trial	Secukinumab	391	Time to LOR (Week 104)	Q2W: 283 days vs placebo 239 days, Q4W: 365 days vs placebo 171 days	COVID-19, Recurrence of HS, nasopharyngitis
Mansilla-Polo, et al. (25)	2025	Multicenter retrospective study	Bimekizumab	84	HiSCR50, IHS4-55 (Week 24)	HiSCR50: 55.95%; IHS4-55: 60.47%	Oral candidiasis
Avallone, et al. (26)	2026	Multicenter retrospective cohort study	Secukinumab	77	HiSCR50, IHS4-55 (Week 52)	HiSCR50: 36.2%; IHS4-55: 55.6%	One case of paradoxical psoriasis
Sayed CJ, et al. (27)	2026	Open-label extension study	Bimekizumab	446	HiSCR50/75/90/100 (Year 2)	HiSCR50:85.4%, HiSCR75:77.1%, HiSCR90:57.6%, HiSCR100:44.2%	Oral candidiasis

RCT, randomized controlled trial; HiSCR, hidradenitis suppurativa clinical response; HS-PGA, hidradenitis suppurativa physician’s global Assessment scale; IHS4, international hidradenitis suppurativa severity score System; AE, adverse events; DLQI, dermatology life quality index; Q2W, every 2 weeks; Q4W, every 4 weeks; LOR, loss of response; COVID-19, coronavirus disease.

Notably, pharmacoeconomic considerations were actively incorporated into the treatment decisions for this patient. When treatment was initiated in March 2025, secukinumab had already been approved for HS in China but was not yet covered by national insurance. It was included in the insurance scheme in September of the same year, but only for adults. Vunakizumab has only been approved for adult psoriasis and ankylosing spondylitis in China, and its use in HS is out-of-pocket. Thus, both drugs require self-payment. After thorough discussion, we selected vunakizumab because of its lower annual out-of-pocket costs. In the Chinese healthcare system, when expected efficacy and safety of drugs are comparable, we choose the medication with better cost-effectiveness based on the patient’s specific situation, as this helps improve treatment adherence and long-term benefits for patients with chronic diseases.

Furthermore, the 5-year delay in diagnosis highlights the diagnostic challenges of HS. Studies have shown an average delay of 10 years between disease onset and diagnosis (1). HS is often misdiagnosed as bacterial folliculitis, recurrent furunculosis, carbuncle, or other acute bacterial infectious skin diseases, leading to the prescribing of numerous ineffective antibiotic treatments, delaying disease management. Some patients may also be misdiagnosed with pilonidal sinus, acne conglobata, perianal Crohn’s disease, or even pyoderma gangrenosum (1). To clarify the diagnostic basis for this patient, in addition to focusing on the core diagnostic features of typical location, typical lesions, and typical disease course, we performed a thorough differential diagnosis. Folliculitis typically presents as superficial, small pustules involving only a single hair follicle; furuncles and carbuncles involve single or multiple hair follicles and surrounding tissue, presenting as red, swollen, firm, painful nodules that may develop pustules as they enlarge; neither condition exhibits the deep inflammatory nodules, abscesses, and interconnected sinus tracts seen in this case. Although recurrent furunculosis recurs frequently, it does not result in tunnel or scar formation after healing, which are typical manifestations of HS. Pilonidal sinus is usually confined to the midline sacrococcygeal region and is associated with hair embedding, whereas the patient’s lesions were distributed bilaterally on the buttocks, not midline, and ultrasound showed no evidence of hair or other foreign bodies. Acne conglobata, although presenting with deep nodules and cysts, primarily involves sebaceous gland-rich areas such as the face, chest, and back, with a distinctly different anatomical predilection. Perianal Crohn’s disease may present with perianal abscesses, ulcers, fistulas, or scarring, but is often accompanied by gastrointestinal symptoms (abdominal pain, diarrhea, weight loss, etc.), which are absent in HS. Finally, pyoderma gangrenosum typically presents as rapidly progressive painful ulcers with undermined, violaceous borders and pathergy, rather than the chronic, suppurative nodules and tunnels characteristic of HS. In summary, clinicians should note that that for recurrent painful nodules, abscesses, or tunnels in skin fold areas, careful differential diagnosis is necessary, and a high index of suspicion for HS should be maintained to enable early recognition and intervention, as this is crucial for improving HS prognosis.

This is a single-case report with limited follow-up; thus, no conclusions can be drawn regarding the long-term efficacy or rare adverse events of vunakizumab. A prospective multicenter single-arm study of vunakizumab for moderate-to-severe HS has been initiated in China to provide higher-level evidence. This case report, providing real-world data with a focus on pharmacoeconomics, may serve as a reference for clinicians facing multiple treatment options.

## 4 Conclusion

We report a case of successful treatment of moderate-to-severe HS with vunakizumab. This case suggests that vunakizumab may be a potential therapeutic option for HS warranting further investigation. Moreover, the treatment decision incorporated pharmacoeconomic considerations, with selection of a more cost-effective drug prior to insurance coverage, reflecting a real-world individualized strategy that balances efficacy and cost. In addition, the 5-year diagnostic delay highlights the need for improved early recognition of HS. As a single case report, these findings require validation in larger, long-term prospective studies.

## Patient perspective

The patient reported a significant improvement in quality of life following clinical amelioration, enabling greater focus on academic pursuits.

## Data Availability

The original contributions presented in the study are included in the article/supplementary material, further inquiries can be directed to the corresponding author/s.

## References

[1] Zouboulis CC Bechara FG Fritz K Goebeler M Hetzer FH Just E . S2k guideline for the treatment of hidradenitis suppurativa / acne inversa - short version. J Dtsch Dermatol Ges. (2024) 22:868–89. doi: 10.1111/ddg.15412 38770982

[2] Wolk K Join-Lambert O Sabat R . Aetiology and pathogenesis of hidradenitis suppurativa. Br J Dermatol. (2020) 183:999–1010. doi: 10.1111/bjd.19556 33048349

[3] Ingram JR Collier F Brown D Burton T Burton J Chin MF . British Association of Dermatologists guidelines for the management of hidradenitis suppurativa (acne inversa) 2018. Br J Dermatol. (2019) 180:1009–17. doi: 10.1111/bjd.17537 30552762

[4] Zouboulis CC Desai N Emtestam L Hunger RE Ioannides D Juhász I . European S1 guideline for the treatment of hidradenitis suppurativa/acne inversa. J Eur Acad Dermatol Venereol. (2015) 29:619–44. doi: 10.1111/jdv.12966 25640693

[5] Gao JL Otto TS Porter ML Kimball AB . Hidradenitis suppurativa: new targets and emerging treatments. Am J Clin Dermatol. (2024) 25:765–78. doi: 10.1007/s40257-024-00880-1 39060744

[6] Pinto Salgueiro G Yilmaz O Nogueira M Torres T . Interleukin-17 inhibitors in the treatment of hidradenitis suppurativa. BioDrugs. (2025) 39:53–74. doi: 10.1007/s40259-024-00687-w 39604776 PMC11750882

[7] Schlapbach C Hänni T Yawalkar N Hunger RE . Expression of the IL-23/Th17 pathway in lesions of hidradenitis suppurativa. J Am Acad Dermatol. (2011) 65:790–8. doi: 10.1016/j.jaad.2010.07.010 21641076

[8] Kelly G Hughes R McGarry T van den Born M Adamzik K Fitzgerald R . Dysregulated cytokine expression in lesional and nonlesional skin in hidradenitis suppurativa. Br J Dermatol. (2015) 173:1431–9. doi: 10.1111/bjd.14075 26282467

[9] Moran B Sweeney CM Hughes R Malara A Kirthi S Tobin AM . Hidradenitis suppurativa is characterised by dysregulation of the Th17: Treg cell axis, which is corrected by anti-TNF therapy. J Invest Dermatol. (2017) 137:2389–95. doi: 10.1016/j.jid.2017.05.033 28652108

[10] Holcomb ZE Porter ML Kimball AB . A safety review of biologic therapies for the management of hidradenitis suppurativa and unmet needs. Expert Opin Drug Saf. (2021) 20:1147–61. doi: 10.1080/14740338.2021.1924147 33910441

[11] Kelhälä HL Palatsi R Fyhrquist N Lehtimäki S Väyrynen JP Kallioinen M . IL-17/Th17 pathway is activated in acne lesions. PloS One. (2014) 9:e105238. doi: 10.1371/journal.pone.0105238 25153527 PMC4143215

[12] Van Straalen KR Prens EP Gudjonsson JE . Insights into hidradenitis suppurativa. J Allergy Clin Immunol. (2022) 149:1150–61. doi: 10.1016/j.jaci.2022.02.003 35189127

[13] Kimball AB Loesche C Prens EP Bechara FG Weisman J Rozenberg I . IL-17A is a pertinent therapeutic target for moderate-to-severe hidradenitis suppurativa: combined results from a pre-clinical and phase II proof-of-concept study. Exp Dermatol. (2022) 31:1522–32. doi: 10.1111/exd.14619 35638561 PMC9804780

[14] Glatt S Jemec GBE Forman S Sayed C Schmieder G Weisman J . Efficacy and safety of bimekizumab in moderate to severe hidradenitis suppurativa: a phase 2, double-blind, placebo-controlled randomized clinical trial. JAMA Dermatol. (2021) 157:1279–88. doi: 10.1001/jamadermatol.2021.2905 34406364 PMC8374742

[15] National Library of Medicine . A Randomised, Double-Blind, Placebo Controlled, Multiple Dose Study to Evaluate the Clinical Efficacy, Safety, Tolerability, Dose Relation, Pharmacokinetics and Pharmacodynamics of CJM112 in Moderate to Severe Chronic Hidradenitis Suppurativa Patients (Accessed May 28, 2024).

[16] Osorio-Gómez GF Ortiz-Álvarez J Diaz-Ceca D Guijarro-Sánchez C Conejo-Mir Sánchez J Fernández Crehuet P . Brodalumab for moderate-severe hidradenitis suppurativa: an open-label multicentric cohort study in real clinical practice. Australas J Dermatol. (2024) 65:254–9. doi: 10.1111/ajd.14267 38597096

[17] Kearney N Hughes R Kirby B . Treatment of hidradenitis suppurativa with Brodalumab in biologic treatment failures: experiences from a specialty clinic. Clin Exp Dermatol. (2023) 48:790–2. doi: 10.1093/ced/llad130 37017188

[18] Kimball AB Jemec GBE Alavi A Reguiai Z Gottlieb AB Bechara FG . Secukinumab in moderate-to-severe hidradenitis suppurativa (SUNSHINE and SUNRISE): week 16 and week 52 results of two identical, multicentre, randomised, placebo-controlled, double-blind phase 3 trials. Lancet. (2023) 401:747–61. doi: 10.1016/S0140-6736(23)00022-3 36746171

[19] Kimball AB Jemec GBE Sayed CJ Kirby JS Prens E Ingram JR . Efficacy and safety of bimekizumab in patients with moderate-to-severe hidradenitis suppurativa (BE HEARD I and BE HEARD II): two 48-week, randomised, double-blind, placebo-controlled, multicentre phase 3 trials. Lancet. (2024) 403:2504–19. doi: 10.1016/S0140-6736(24)00101-6 38795716

[20] Mansilla-Polo M Pons-Benavent M Fernández-Crehuet P Vilarrasa E Albanell-Fernández C Morales-Tedone E . Real-world effectiveness and safety of bimekizumab for hidradenitis suppurativa: an ambispective observational study. Australas J Dermatol. (2024) 65:e198-202. doi: 10.1111/ajd.14339 38895808

[21] Haselgruber S Fernández-Crehuet-Serrano P Fernández-Ballesteros MD Padial-Gómez A Hernández-Rodríguez JC Ortiz-Álvarez J . Insights into the window of opportunity and outcome measures in patients with moderate to severe hidradenitis suppurativa treated with secukinumab: a real-world study. Dermatol Ther (Heidelb). (2024) 14:1875–90. doi: 10.1007/s13555-024-01209-w 38896382 PMC11264522

[22] National Library of Medicine, Phase A . 2b Pivotal Study to Evaluate the Efficacy and Safety of Izokibep in Subjects With Moderate to Severe Hidradenitis Suppurativa (Accessed May 28, 2024). ClinicalTrials.gov.

[23] Kimball AB Kirby B Bechara FG . Efficacy and safety of the IL-17A- and IL-17F-inhibiting Nanobody® sonelokimab in patients with moderate-to-severe hidradenitis suppurativa (HS); week 24 results from the phase 2 MIRA trial (2024). San Diego, California (Accessed 8-12 March, 2024).

[24] Kimball AB Bechara FG Badat A Giamarellos-Bourboulis EJ Gottlieb AB Jemec GBE . Long-term efficacy and safety of secukinumab in patients with moderate-to-severe hidradenitis suppurativa: week 104 results from the SUNSHINE and SUNRISE extension trial. Br J Dermatol. (2025) 192:629–40. doi: 10.1093/bjd/ljae469 39611771

[25] Mansilla-Polo M Pons-Benavent M Fernández-Crehuet P Vilarrasa E Albanell-Fernández C Morales-Tedone E . Safety and efficacy of bimekizumab in patients with moderate-to-severe hidradenitis suppurativa: a multicenter retrospective cohort study. Actas Dermosifiliogr. (2026) 117:104531. doi: 10.1016/j.ad.2025.104531 41354335

[26] Avallone G Roccuzzo G Valtellini L Moltrasio C Genovese G Repetto F . Long-term outcomes of secukinumab in moderate-to-severe hidradenitis suppurativa: a multicentre cohort study. J Cutan Med Surg. (2026) 17:12034754261418237. doi: 10.1177/12034754261418237 41841557

[27] Sayed CJ Kirby B Garg A Naik HB Kimball AB Zouboulis CC . Bimekizumab demonstrated a favorable safety profile and high levels of efficacy with up to 2 years of treatment in patients with moderate to severe hidradenitis suppurativa: pooled results from two phase 3 randomized, controlled trials and their open-label extension. J Am Acad Dermatol. (2026) 94:867–78. doi: 10.1016/j.jaad.2025.11.031 41248770

